# Experimental evaluation of oil recovery mechanism using a variety of surface-modified silica nanoparticles: Role of *in-situ* surface-modification in oil-wet system

**DOI:** 10.1371/journal.pone.0236837

**Published:** 2020-07-30

**Authors:** Muhammad Adil, Hasnah Mohd Zaid, Faizan Raza, Mohd Arif Agam

**Affiliations:** 1 Department of Fundamental and Applied Sciences, Universiti Teknologi PETRONAS, Tronoh, Perak, Malaysia; 2 Department of Chemical Engineering, NED University of Engineering and Technology, Karachi, Sindh, Pakistan; 3 Department of Sciences, Universiti Tun Hussein Onn Malaysia, Panchor, Johor Darul Takzim, Malaysia; Texas A&M University at Qatar, QATAR

## Abstract

Recent developments propose renewed use of surface-modified nanoparticles (NPs) for enhanced oil recovery (EOR) due to improved stability and reduced porous media retention. The enhanced surface properties render the nanoparticles more suitable compared to bare nanoparticles, for increasing the displacement efficiency of waterflooding. However, the EOR mechanisms using NPs are still not well established. This work investigates the effect of in-situ surface-modified silica nanoparticles (SiO_2_ NPs) on interfacial tension (IFT) and wettability behavior as a prevailing oil recovery mechanism. For this purpose, the nanoparticles have been synthesized via a one-step sol-gel method using surface-modification agents, including Triton X-100 (non-ionic surfactant) and polyethylene glycol (polymer), and characterized using various techniques. These results exhibit the well-defined spherical particles, particularly in the presence of Triton X-100 (TX-100), with particle diameter between 13 to 27 nm. To this end, SiO_2_ nanofluids were formed by dispersing nanoparticles (0.05 wt.%, 0.075 wt.%, 0.1 wt.%, and 0.2 wt.%) in 3 wt.% NaCl to study the impact of surface functionalization on the stability of the nanoparticle suspension. The optimal stability conditions were obtained at 0.1 wt.% SiO_2_ NPs at a basic pH of 10 and 9.5 for TX-100/ SiO_2_ and PEG/SiO nanofluids, respectively. Finally, the surface-treated SiO_2_ nanoparticles were found to change the wettability of treated (oil-wet) surface into water-wet by altering the contact angle from 130° to 78° (in case of TX-100/SiO_2_) measured against glass surface representing carbonate reservoir rock. IFT results also reveal that the surfactant treatment greatly reduced the oil-water IFT by 30%, compared to other applied NPs. These experimental results suggest that the use of surface-modified SiO_2_ nanoparticles could facilitate the displacement efficiency by reducing IFT and altering the wettability of carbonate reservoir towards water-wet, which is attributed to more homogeneity and better dispersion of surface-treated silica NPs compared to bare-silica NPs.

## Introduction

Nanoparticles have emerged as promising materials in the light of a decline in production from existing oil fields and the increasing difficulty of extracting hydrocarbons through conventional EOR methods, including surfactant and polymer flooding. Surfactant flooding has constraints as they cannot be maintained over a long period, especially in the presence of high temperature and high salinity [1]. Similarly, the polymers precipitated at the elevated temperature, particularly in the presence of divalent cations (e.g. Mg^2+^, Ca^2+^) due to a bridging effect [2]. The robustness and activity of surfactant/polymer can, however, be considerably improved by introducing surface-modified nanoparticles [3–7]. For this purpose, silica nanoparticles have been widely considered [8–10]. They are not only environmentally friendly particularly in high reservoir temperature and pressure [11,12], but once incorporated into the subsurface, they can build or split emulsions or alter the wettability of porous media [13,14] and thereby improving oil recovery. The studies aiming at explaining the oil recovery due to silica NPs injection have suggested multiple EOR mechanisms such as; i) IFT reduction and wettability alteration [9,14–16], ii) structural disjoining pressure [17], iii) formation of in- situ emulsions [18–20], and iv) Pore blocking and microscopic flow diversion [21–23]. Silica NPs adsorb along with the oil/water interface to lower the IFT between oil and water and to change the wettability to more water-wet condition [13,24], which are the two most proposed EOR mechanisms. In addition, silica nanoparticles can be surface modified to satisfy specific reservoir environments [25,26], by solving some of the issues found with bare silica NPs.

Silica NPs, with an attached surfactant/polymer to its surface, form a unique class of nanomaterials for EOR that could be superior to unmodified NPs due to enhanced properties such as stability, emulsion stabilization, low porous media retention, etc. [14,27]. These are classified as functionalized NPs, which have proved to be effective for different reservoir conditions [28]. Various studies have shown that NPs dispersed in surfactant solutions can increase the oil recovery through synergistic effects relative to the surfactant flooding alone [29–31]. Zargartalebi et al. [32] demonstrated that the use of hydrophilic silica NPs in addition to sodium dodecyl sulfate (SDS) decreased IFT and improved oil recovery from sandstone rocks. The finding obtained were in accordance with those observed by Ahmadi and Shadizadeh [33]. Nwidee et al. [34], showing that the dispersion of the surfactant-coated NPs changed the system’s wettability to water-wet condition. Behzadi and Mohammadi [35] have shown that polymer-functionalized silica NPs can reduce IFT between oil/water and alter the wettability of the oil-wet glass micromodel to more water-wet, leading to improved oil recovery than bare silica NPs. Experimental studies performed by Choi et al. [36] showed that 74.1% of the oil was recovered when polymer-coated silica NPs were injected into water-wet core samples, in comparison to 68.9% of original oil in place (OOIP) from waterflooding and 72.7% of OOIP from bare silica NPs. Bila et al. [37] recently tested numerous polymer-functionalized silica NPs and recorded a maximum oil recovery of 5.2% of OOIP after waterflooding. Like Choi et al. [36], the oil recovery was a result of reduction in interfacial tension and change in wettability to more water-wet state.

Based on the above studies, surface-modified NPs can provide a pathway for EOR, however, more studies are required to further understand and verify the contribution of each of the proposed NP’s mechanisms for oil recovery. This is crucial to attaining optimum conditions for a significant increase in oil recovery to make nanotechnology robust for field applications. For this purpose, in-situ surface-modified silica NPs were synthesized using the sol-gel method assisted by reverse micelle microemulsion in the presence of nonionic surfactant Triton X-100 and polyethylene glycol (PEG-20000). The result is a simple and easy one-step method for self-assembling structurally well-defined silica NPs of the desired size. The silica nanoparticles’ structure, morphology, and size were characterized through field-emission scanning electron microscopy (FESEM), energy dispersive X-ray spectroscopy (EDX), X-ray diffraction (XRD), infrared spectroscopy (FT-IR), and Brunauer-Emmett-Teller (BET). The stability of these nanoparticles was then evaluated in brine by observing their sedimentation behavior using the visualization method, along with the measurement of average hydrodynamic size using dynamic light scattering technique. These measurements were conducted to determine the stability of the nano-suspension which may be affected by the pH of aqueous suspension and the weight percentage of the suspended nanoparticles. Finally, IFT and contact angle measurements were conducted to evaluate the performance of stable nano-silica dispersions on oil recovery mechanisms under oil-wet conditions.

## Materials and methods

### Synthesis of silica NPs

Silica NPs were synthesized using the sol-gel process in three different ways, where all the reagents were used without further purification.

### Bare silica NPs

The bare silica NPs were synthesized using the modified Stöber method with the experimental parameters tabulated in Table 1. A quantity of deionized water (≥18 MΩ) was first added in absolute ethanol (99%, VWR chemicals) under ultrasonic agitation at room temperature for 10 min. Then, tetraethylorthosilicate (TEOS, Merck) was poured into the reaction mixture with a feed rate of 0.2 mL min^-1^ in order to hydrolyze TEOS in the ultrasonic bath. After 1.5 hours, ammonia (25%, Merck) was added to the reaction media at a feed rate of 0.01 mL min^-1^. Sonication lasted for 3 hours until the delicate gel formed. The gel was then centrifuged and washed with ethanol and distilled water (3 × 10 min, 6000 rpm), before it was dried at 80°C for 24 hours in a drying oven. Lastly, the samples were calcined at 500°C for 2 hours to produce silica nanoparticles.

**Table 1:** Experimental parameters used in the synthesis of silica NPs.

Parameter	Sample 1	Sample 2
TEOS (mol L^-1^)	0.58	0.80
NH_3_ (mol L^-1^)	0.6	1.87
[H_2_O]/[TEOS]	28.8	37.0
Temperature (°C)	25‒50	25‒50

#### Silica/TX-100 NPs

The surfactant coated silica nanoparticles were synthesized using the sol-gel process aided by reverse micelle microemulsion, employing tetraethylorthosilicate (TEOS, Merck) as a silica precursor, Triton X-100 (Sigma-Aldrich) as a non-ionic surfactant, methanol (98%, Merck) as a co-surfactant, Cyclohexane (99.5%, Merck) as an oil phase, ammonia (25%, Merck) as a pH adjuster and deionized water (≥18 MΩ). Surfactant, co-surfactant, and oil phase are first combined at room temperature to form a microemulsion at 700 rpm for 15 min. Two molar ratios (4.5 and 7.6) of [Methanol]/[TX-100] were chosen for this study, while the molar ratio of [H_2_O]/[TX-100] and [H_2_O]/[TEOS] were fixed at 9.2 and 59.1, respecitvely. Upon the generation of microemulsion, the medium is modified to basic pH (10 and 12) with a solution of ammonium hydroxide to catalyze the polymerization reactions required to grow the silica nanoparticles that are produced in the next step. Finally, TEOS is applied to facilitate the hydrolysis and condensation of the silica molecules inside the micelle. The synthesis was fixed at 2 hours [38] in order to obtain the small size nanoparticles by reducing the nucleation period. The microemulsion then was broken with the introduction of ethanol (VWR chemicals). The mixture was centrifuged (3 × 10 min, 6000 rpm) and washed with ethanol and water to recover the nanoparticles by removing the residual amount of surfactant and co-surfactant still present on the nanoparticles. After that, the particles were oven-dried at 80°C for overnight to obtain the dry nano-silica particles.

#### Silica/PEG-20000 NPs

A typical reaction solution, comprised of PEG molecular weight (MW) 20000 monomethyl ether (Sigma-Aldrich), methanol (98%, Merck), ammonium hydroxide (25%, Merck) and tetraethylorthosilicate (TEOS, Merck), was employed in this section The preparation steps for PEGylated silica NPs are as follows: PEG polymer of three weight concentrations (0.1, 0.01, and 0.001 g) was mixed in a combined solution of ammonia (3 mL) and methanol (24 mL). The mixture becomes transparent when stirred, and TEOS (0.1 mL) was added dropwise to start the hydrolysis reaction. Then, the mixture was agitated vigorously for 3 hours at room temperature before the reaction was stopped. The reaction was then stopped by the addition of a moderate quantity of ethanol to the solution. The mixture was centrifuged (3 × 10 min, 6000 rpm) and further rinsed with ethanol and water to ensure all unreacted PEG and TEOS from the silica nanoparticles had been removed. After that, the samples were dried at 80°C to produce PEGylated silica NPs as the final product.

### Characterization of silica NPs

The size and shape of the as-synthesized nanoparticles were measured with a field emission scanning electron microscope (FESEM, Zeiss Supra 55VP), equipped with energy dispersive X-ray spectroscopy (EDX) for elemental analysis. The term particle size used in this article refers to the mean diameter of the silica nanoparticles. The average diameter was estimated for near-spherical particles. The XRD pattern of SiO_2_ nanoparticles was obtained using X-ray diffraction (XRD, Bruker D8) with CuK_α_ (λ = 1.5406 Å) radiation source. The measurements were conducted between a 2θ of 10 ‒ 90°±0.1 at an accelerated voltage of 40 kV and current 40 mA. The composition of the silica samples was analyzed using Fourier Transform Infrared (FT-IR) spectrometer (Perkin Elmer) in the frequency range of 4000–400 cm^-1^, where the measured transmittance was within ±0.1%T. On the other hand, ASAP 2020 adsorption analyzer (Micromeritics) was used to measure the surface area and porosity by using nitrogen adsorption–desorption (77 K) and the Brunauer-Emmett-Teller (BET) algorithm. The nano-samples were degassed overnight at 250°C under vacuum (10^−3^ mmHg). Assuming that the silica nanoparticles are homogeneous, non-porous, and spherical, the average particle size *D* (nm) was determined using the spherical model equation [39,40]:

$$D=6⁄\rho S_{sp}$$ (1)

where *ρ* is the density of silica nanoparticles (2.0 × 10^6^ gm^-3^ for silica nanoparticles synthesized using wet-synthesis method [41]) and *S*_*sp*_ is the surface area of the spherical nanoparticles.

### Nanofluids preparation

The nanofluids were prepared by simply dispersing the in-situ modified nanoparticles in 3 wt.% brine (equivalent to seawater concentration) to study the effect of pH and nanoparticle concentrations on the dispersion using sedimentation approach as well as zeta potential and hydrodynamic size measurement. NPs with concentration between 0.05–0.20 wt.% were added in brine as the basefluid and agitated magnetically for 1 hour to obtain nano-suspension. In this stage, the pH was modified based on autotitration results to achieve high nanoparticles zeta potential which provides high electrostatic repulsion, resulting in a more uniform and stable nano-suspension. The suspension was then agitated at room temperature in an ultrasonic bath for 30 min to achieve a homogeneous dispersion in the basefluid. The zeta potentials and diameters of the silica NFs were determined using the Zetasizer Nano-ZS dynamic light scattering instrument (Malvern Instruments Inc., UK).

### IFT and wettability measurements

The interfacial tension between crude oil and brine/NFs as aqueous phase was determined by using SVT20N spinning drop video tensiometer (Data Physics) at ambient environment. For this purpose, light crude oil sourced from Miri has been used with a density and viscosity of 0.8481 g/cm^3^ and 5.53 cP, respectively. Meanwhile, each nano-sample was run three times to achieve a consistent value. Since the oil density is smaller than the density of nanofluid, the oil droplet couldn’t stabilize in the nanofluid, rendering it difficult to use the pendant drop method for IFT analysis. The oil droplet volume was maintained in the range of 2–3 mL, while the rotational speed was maintained between 5000–6000 rpm. The formula to calculate IFT is as follows [41]:

$$\sigma =\frac{△\rho \Omega^{2}{\left(D_{app}\right)}^{3}}{8n^{3}J_{D}\left(L/D\right)}$$ (2)

where *s* is the measured IFT (dyn/cm), *Δρ* is the density difference between oil and aqueous phase (g/cm^3^), *Ω* is the rotational rate of the cylinder (s^-1^), *D*_*app*_ is the measured diameter of oil droplet (cm), *n* is the refractive index of the heavy fluid, *D* is the true diameter of the oil droplet (*D = D*_*app*_*/n*), *J*_*D*_ is the correction factor and function of *L/D*, and *L/D* is the aspect ratio.

**Fig 1:**
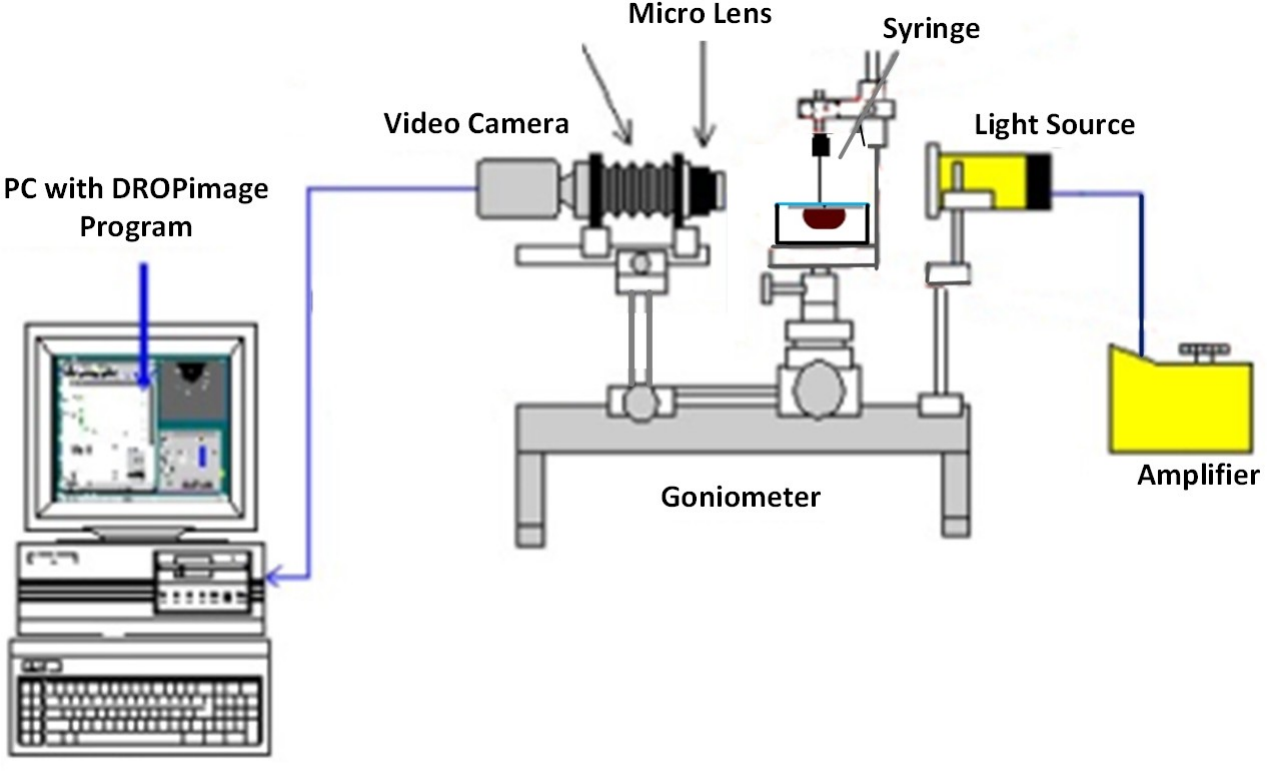
Schematic of the Goniometer for contact angle measurement using the sessile-drop method.

Three runs of contact angle measurement by using the static sessile-drop method were applied to investigate the wetting characteristic of an oil-wet surface. These measurements were performed by employing a Goniometer (Ramé-hart Model 260) in ambient conditions, as shown in Fig 1. For this purpose, small glass plates (7.6 cm × 2.5 cm × 1.3 mm) were used and rendered oil-wet by the following method: the plates were fully soaked for 1 hour in NaOH solution and then thoroughly rinsed with distilled water. Subsequently, they were dried at 200°C in an oven for 15 min. The plates were then saturated between 5–10 min with a solution of 98% toluene and 2% trichloromethylsilane. They were then again rinsed with methanol and dried at 100°C for 1 hour. The initial oil-wetness of treated-glass plates were determined by measuring the contact angle values of deionized water (91°), brine (96°), and crude oil (150°). These modified plates were then immersed in the NFs, consist of brine and silica NPs, in an effort to recover their original water wetness. The glasses plates were aged in nanofluids for approximately 24 hours, followed by drying overnight in a drying oven. This procedure was repeated at different nanoparticle concentrations to determine wettability by contact angle measurement. Next, a droplet of the crude oil (24 ± 0.2 μL) was placed using a J-shaped needle underneath the glass surface placed over a cell filled with silica nanofluid. The measurement was performed before and after aging with the corresponding silica nanofluid solutions. Naturally, the oil droplet moves upward driven by buoyancy force and spread on the surface. The camera was manually adjusted to achieve a focused and magnified image of the drop and the surface, which can be seen on the connected computer. Once the drop-shape profile reached equilibrium, the image of the droplet was captured. DROPimage software then used the side-view profile to measure the three-phase contact angle using the image analysis technique. Finally, an average contact angle was calculated using both sides of each image with the tolerance of ± 5 degrees of contact angle.

## Results and discussion

### Bare silica NPs

The formation of silica NPs from TEOS reactions is generally carried out through the following steps [42]:

1. *Hydrolysis*

$$Si{\left(OC_{2}H_{5}\right)}_{4}+H_{2}O\to Si{\left(OC_{2}H_{5}\right)}_{3}OH+C_{2}H_{5}OH$$ (3)

Water condensation

$$\equiv Si-O-H+H-O-Si\to \equiv Si-O-Si\equiv +H_{2}O$$ (4)

Alcohol condensation

$$\equiv Si-O-C_{2}H_{5}+H-O-Si\equiv \to \equiv Si-O-Si+C_{2}H_{5}OH$$ (5)

**Fig 2:**
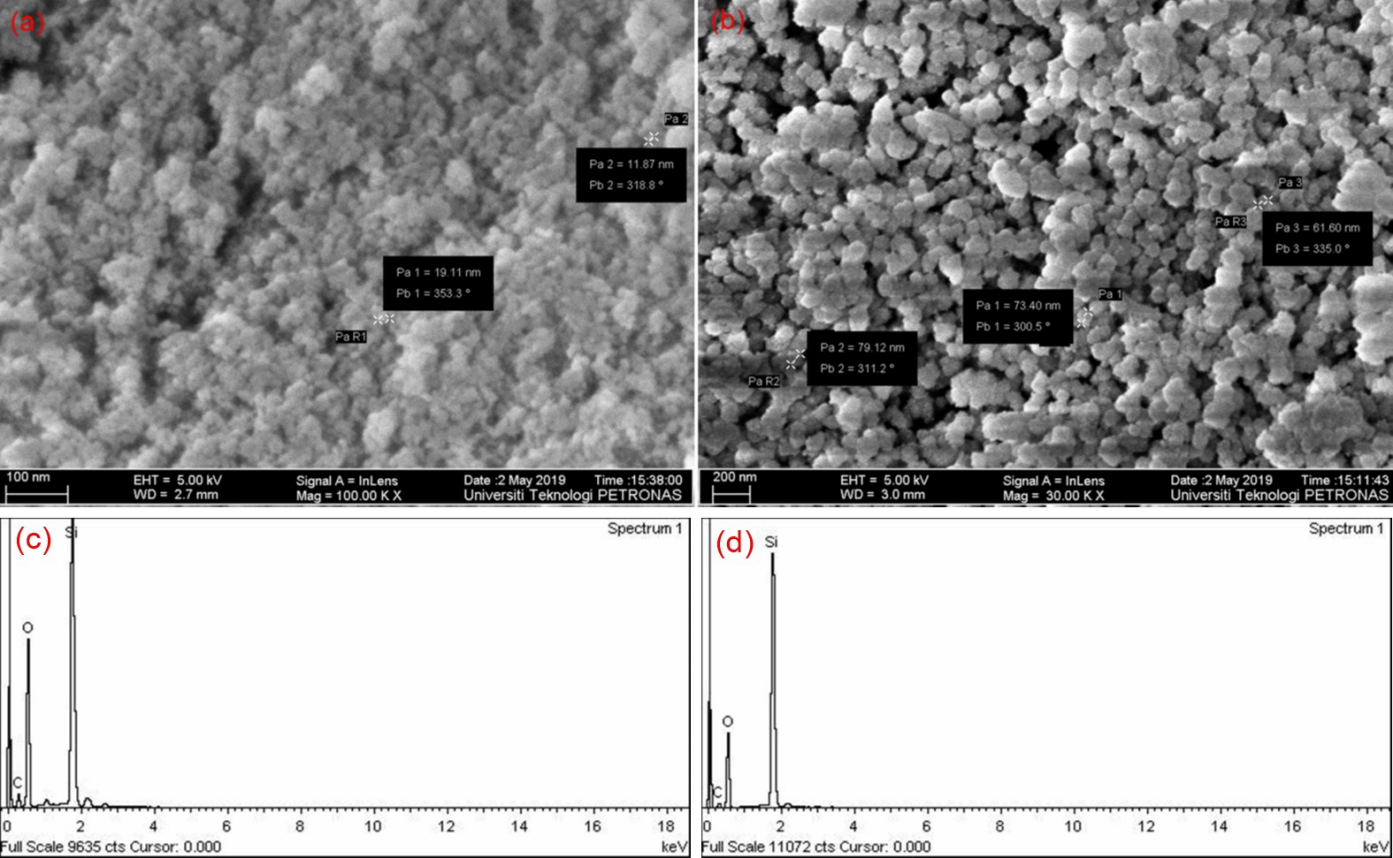
Photomicrographs of FESEM (a, b) and EDX spectrum (c, d) of silica nanoparticles synthesized via sol-gel using 0.58 mol L^-1^ (a, c) and 0.80 mol L^-1^ (b, d) of TEOS.

The production of homogeneous, monodispersed silica nanoparticles is strongly dependent on the reaction conditions. The FESEM images in (Fig 2A and 2B) show the impact of TEOS concentrations on the size of the bare-silica nanoparticles, where the particle diameter increases as the TEOS concentration increases. This observation clearly implies the increase of primary particles during the induction period, i.e. [primary particles] ∝ [TEOS], leading to stable silica nanoparticles. The FESEM images also demonstrate that the nanoparticles were highly aggregated and broadly distributed. For the compositional analysis of silica NPs, EDX measurementis performed and depicted in (Fig 2C and 2D). The EDX shows that the pure Si-O phase is present in the nano sample, with a relatively small amount of carbon. The atomic % of Si and O_2_ of SiO_2_ as shown in (Fig 2C and 2D) are also in accordance with the previous study [43]. Moreover, the atomic % of Si is reduced with the increase in TEOS concentration, attributed to the effect on the kinetics of hydrolysis reaction. This is consistent with FESM results, where the particle size increase with the increase in TEOS concentration.

**Fig 3:**
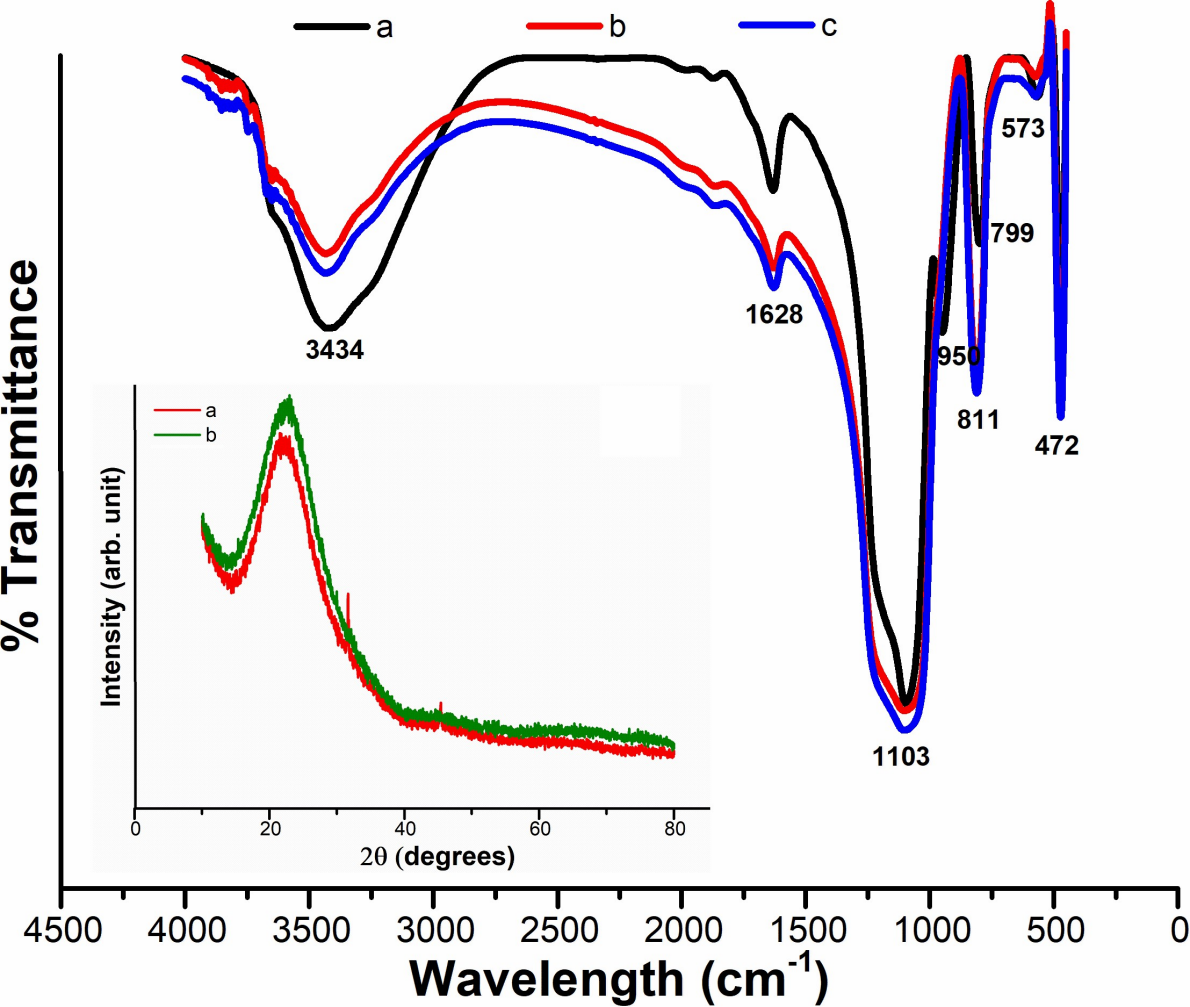
FTIR spectra of (a) as-synthesized bare nano-silica particles, along with calcined nanoparticles at (b) 0.58 mol L^-1^ and (c) 0.80 mol L^-1^ of TEOS including their XRD pattern (the insert figure).

Fig 3 shows FTIR spectra of the as-synthesized and calcined silica nanoparticles. As depicted in the Fig 3A, the peaks corresponding to 950 cm^-1^ (Si–OH, bending vibration) [40] observed in the as-synthesized sample was disappeared in the calcined sample (Fig 3B and 3C), indicating the successful removal process of ethanol. On the other hand, a very intense and broad band can be seen at 1082–1114 cm^-1^ in both samples, which corresponds to asymmetric stretching vibrations of Si–O–Si and depicts the formation of dense silica network [44]. In addition, the other significant infrared vibrations of silica i.e. bending mode of Si–O–Si [45] and stretching of O–H [46] occur at around 472 cm^-1^ and 3434 cm^-1^, respectively. There is another peak observed in the FTIR spectrum at 1628 cm^-1^ which is due to Si–H_2_O flexion [47]. The FTIR study reveals that the silica nanoparticles are of a very hygroscopic nature. On the other hand, the absence of sharp peaks in the XRD pattern (insert Fig 3) proves that there is no crystalline structure in the produced silica NPs. A broad peak at 2θ angle of ~24 is recorded, verifying the amorphous nature of the as-synthesized silica nanoparticles, which is consistent with the previous study [48].

N_2_ adsorption study was performed to determine the properties of the nanoparticles in the solid state. As tabulated in Table 2, surface area *S*_*BET*_ decreases with an increase in the concentration of TEOS due to the agglomeration phenomena that limit the surface area as the nanoparticles join together to create a bulk structure, which is larger than the individual nanoparticles [46]. The high pore size and volume also suggest the existence of open structures due to agglomeration [39]. Such open structures result in the NPs being loosely packed.

**Table 2:** Surface and pore analysis of bare-silica NPs.

Properties	TEOS Concentration (mol L^-1^)	
	0.58	0.80
BET surface area (m^2^ g^-1^)	166.5	44.32
Micropore area (m^2^ g^-1^)	7.38	5.39
Micropore volume (cm^3^ g^-1^)	0.0034	0.0025
Average pore diameter (nm)	14.20	22.55
Average particle size (nm)	18.01	67.68

### Silica/TX-100 NPs

**Fig 4:**
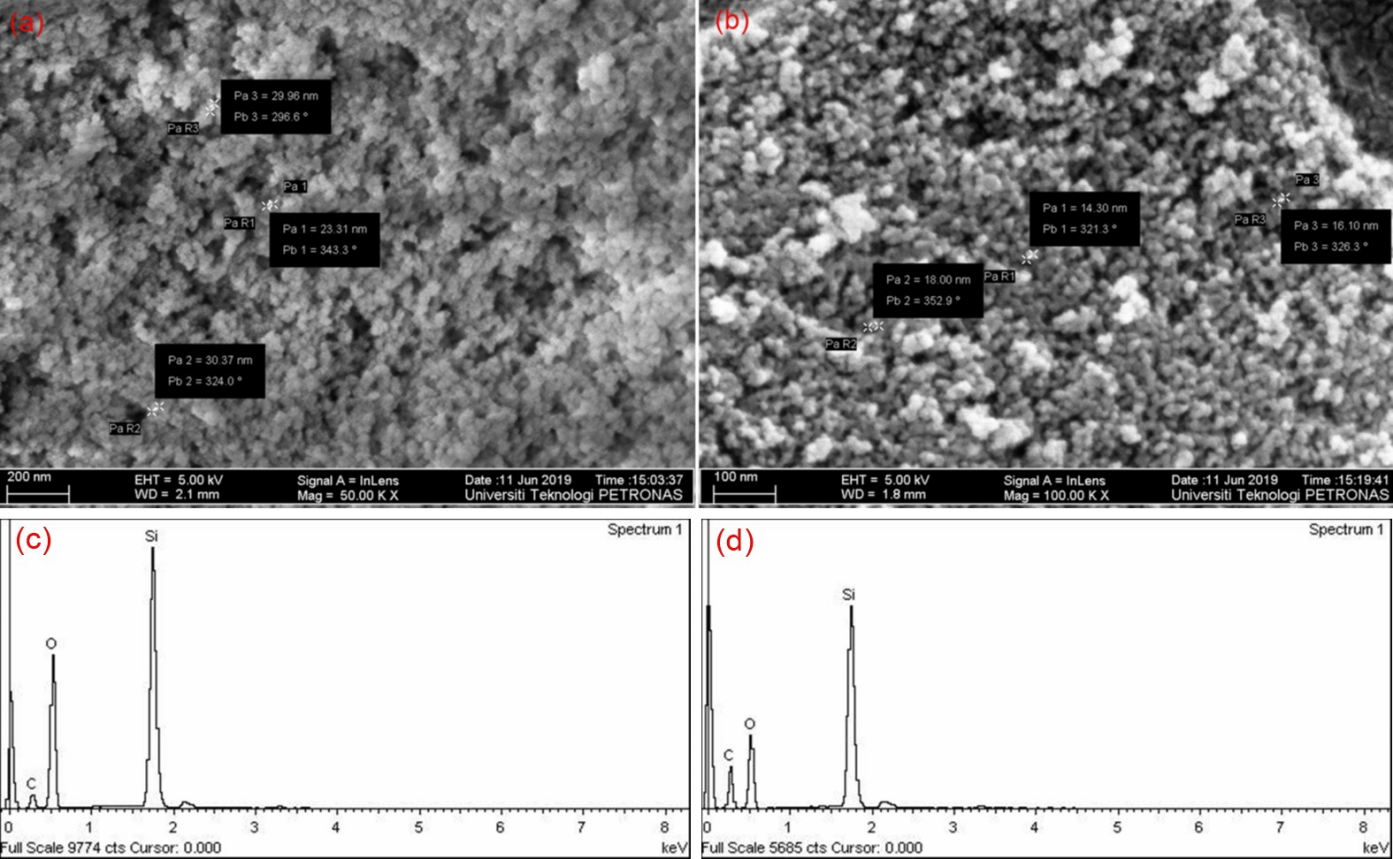
FESEM images (a, b) and EDX spectra (c, d) of surface-modified silica nanoparticles synthesized via sol-gel at varying [Methanol]/ [TX-100] ratio of 4.5 (a, c) and 7.6 (b, d) at pH = 10.

(Fig 4A and 4B) shows that the SiO_2_ NPs have a particle size of (a) 27.9 ± 3.6 nm and (b) 13.1 ± 3.2 nm, when the molar ratio of [Methanol]/[TX-100] changes from 4.5 to 7.6. As the methanol volume increased, the particle size decreases, and they also become more dispersed. This is due to the presence of a smaller amount of Triton X-100 in the micelles, compared to the methanol which results in a drop size being smaller for the availability of polar groups of TX-100 to join methanol groups. The particle size, on the other hand, decreases with the increase in the volume of methanol as more nuclei develop within the silicon micelle, resulting in smaller particle sizes. Meanwhile, EDX spectra of the surfactant-modified silicas were presented in (Fig 4C and 4D), and compared to study the influence of molar ratio of [Methanol]/[TX-100] in the silica matrix. The EDX spectra of surfactant-modified SiO_2_ NPs clearly show the presence of carbon (C). However, the concentration of C noticeably increases to 38% at [Methanol]/[TX-100] = 7.6, which confirmed that TX-100 was more thoroughly incorporated into silica via sol-gel synthesis [38].

**Fig 5:**
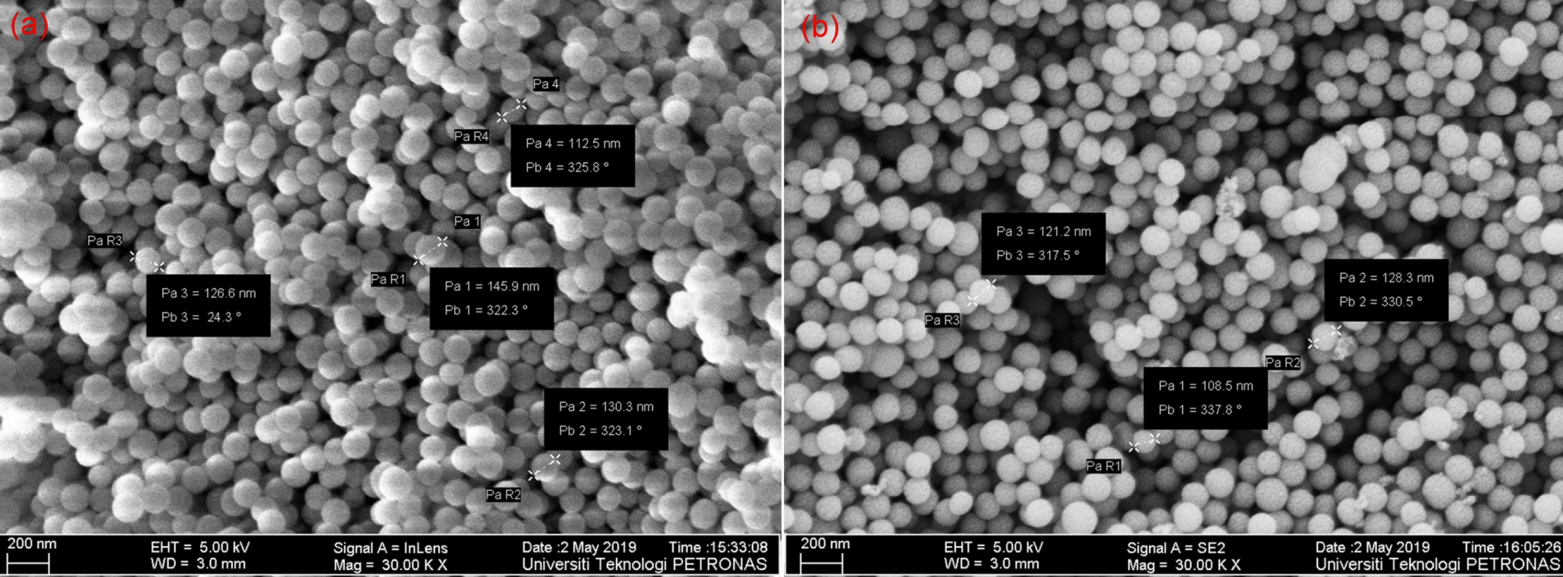
FESEM images of silica nanoparticles synthesized by varying [Methanol]/ [TX-100] a) 4.5 and b) 7.6 at pH = 12 having a corresponding particle diameter of a) 128.8 ± 11.8 nm and b) 119.3 ± 8.1 nm.

The change in pH of the system causes a change in the molar ratio of water-surfactant, which influences not only the particle size but also their morphology (as shown in Fig 5). In this case, pH value was increased from 10 to 12 by the addition of ammonium hydroxide. At higher pH values, larger particle sizes (in the range of nanometers) with well-defined spherical morphology and monodispersity are obtained. The explanation behind the change in particle size, when the pH of the system varies, can be the speed of polymerization reaction. To change the pH value from 7.8 (pH_i_) to 12, a greater amount of ammonium hydroxide is required, producing a large amount of OH and a higher rate of hydrolysis. This contributes to a little number of nuclei produced that form larger nanoparticles [49].

**Fig 6:**
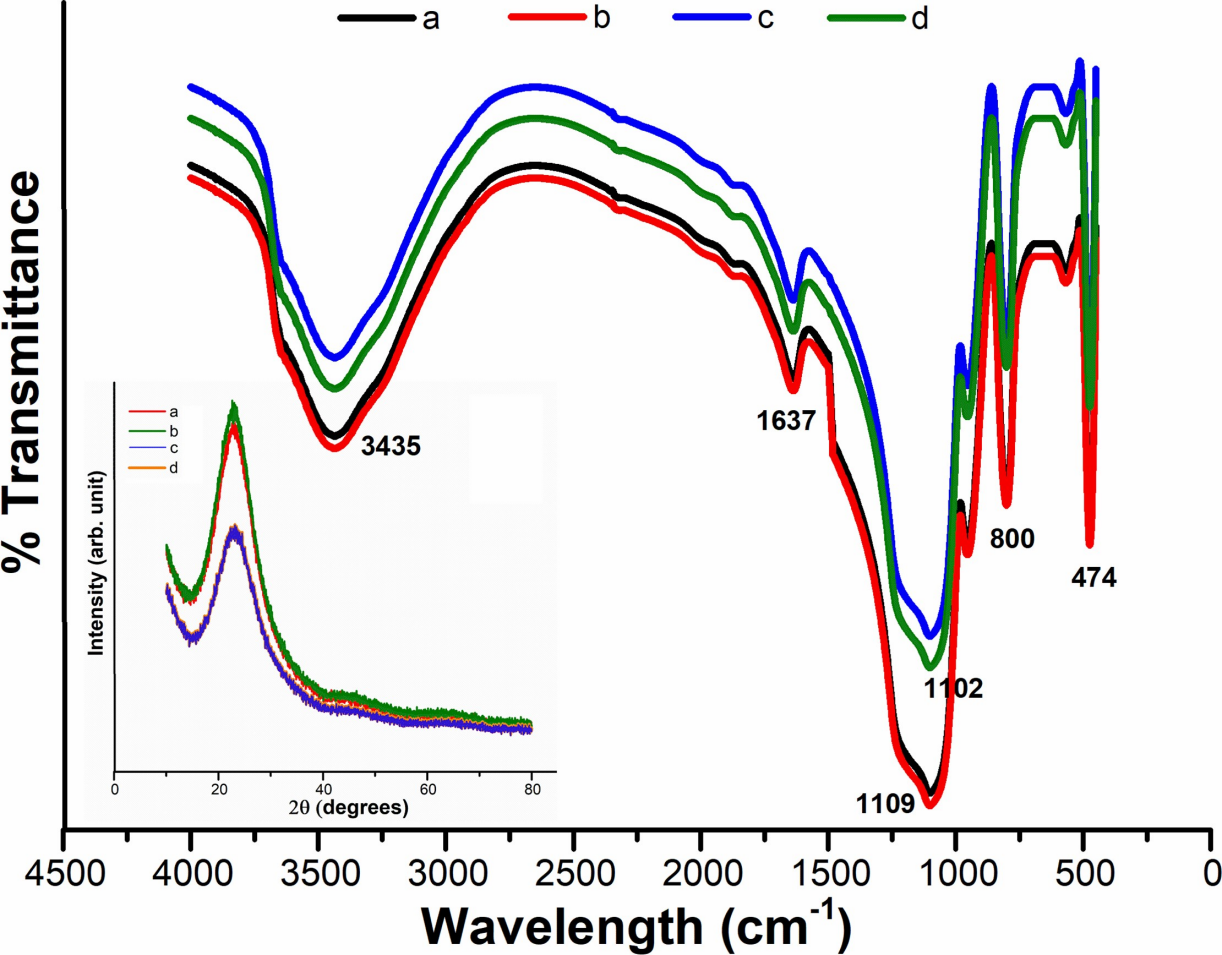
FTIR spectra along with the corresponding XRD pattern (the insert figure) of silica NPs synthesized using molar ratio of [Methanol]/ [TX-100]: a) 4.5, b) 7.6 at pH = 10 and c) 4.5 and d) 7.6 at pH = 12.

A representative FTIR spectrum of SiO_2_ nanoparticles synthesized using different molar ratio of [Methanol]/[TX-100] at pH 10 and 12 is shown in Fig 6. The very intense and broad band that appears at ~1102 and 474 cm^-1^ is assigned to extension and flexural vibrations of Si—O—Si bonds [44], which indicates the formation of a dense silica network. On the other hand, ~800 cm^-1^ absorption band comes from the vibration of (SiO4) tetrahedrons, while the absorption bands at ~3435 and 1637 cm^-1^ originate from O—H bonding vibration of adsorbed molecular water. FTIR spectra also revealed that, as the particle size is decreased, the band at 1109 cm^-1^ was slightly moved to lower wave number. This finding indicates that the local bonding structures of Si and O atoms shift for relatively small nanoparticles [50,51]. On the other hand, XRD patterns of silica nanoparticles shown a typical broad halo in Fig 6 (the insert image), which indicates the amorphous nature of as-synthesized nano-SiO_2_ by sol-gel technique. These results are in accordance with the previous studies [52,53].

N_2_ adsorption-desorption study was carried out to analyze the solid-state characteristics of the nanopowder. As shown in Table 3, *S*_*BET*_ increased as the molar ratio [Methanol]/[TX-100] increased. Although, the surface area can decrease readily due to the agglomeration, where nanoparticles join together to create a bulky structure that is larger than individual nanoparticles [54]. On the other hand, pore volume (*V*_*p*_) was found to increase in the order of 7.6 < 4.5, correlating to the increase in capillary condensation. The pores were formed by the voids present between i) the nanoparticles, ii) the nanoparticles and agglomerates and iii) the agglomerates and also within (see Fig 4) [39]. In addition, pore size diameter in Table 3 supports the IUPAC definition of pore size i.e. 2–50 nm, for mesoporosity behavior in the silica [40]. The high volume and pore size of nano-samples reveal the presence of open structures associated with agglomeration [39]. These open structures result in nanoparticles being loosely packed. Both samples revealed micropores within the structure, which might represent the fine pores within the agglomerates.

**Table 3:** Structural and surface properties of nano-silica prepared via a variation in [Methanol]/[TX-100] at pH = 10.

Properties	[Methanol]/[TX-100]	
	4.5	7.6
BET surface area (m^2^ g^-1^)	104.9	236.3
Micropore area (m^2^ g^-1^)	5.39	12.6
Micropore volume (cm^3^ g^-1^)	0.00255	0.00503
Average pore diameter (nm)	22.55	14.26
Average particle size (nm)	28.59	12.69

### Silica/PEG-20000 NPs

In our process of generating spherical PEG-coated silica NPs, PEG and TEOS proceed through the following steps [55]:

Hydrolysis

$$\cdots \;Si-OR+HO{\left(CH_{2}CH_{2}O\right)}_{n}H↔\;\cdots \;Si-O{\left(CH_{2}CH_{2}O\right)}_{n}H+ROH$$ (6)

Condensation

$$\cdots \;Si-OR+HO{\left(CH_{2}CH_{2}O\right)}_{n}H↔\;\cdots \;Si-O{\left(CH_{2}CH_{2}O\right)}_{n}H+ROH$$ (7)

Silica formation

⋯Si−OR+HO−Si↔⋯Si−O−Si+ROH (8)

**Fig 7:**
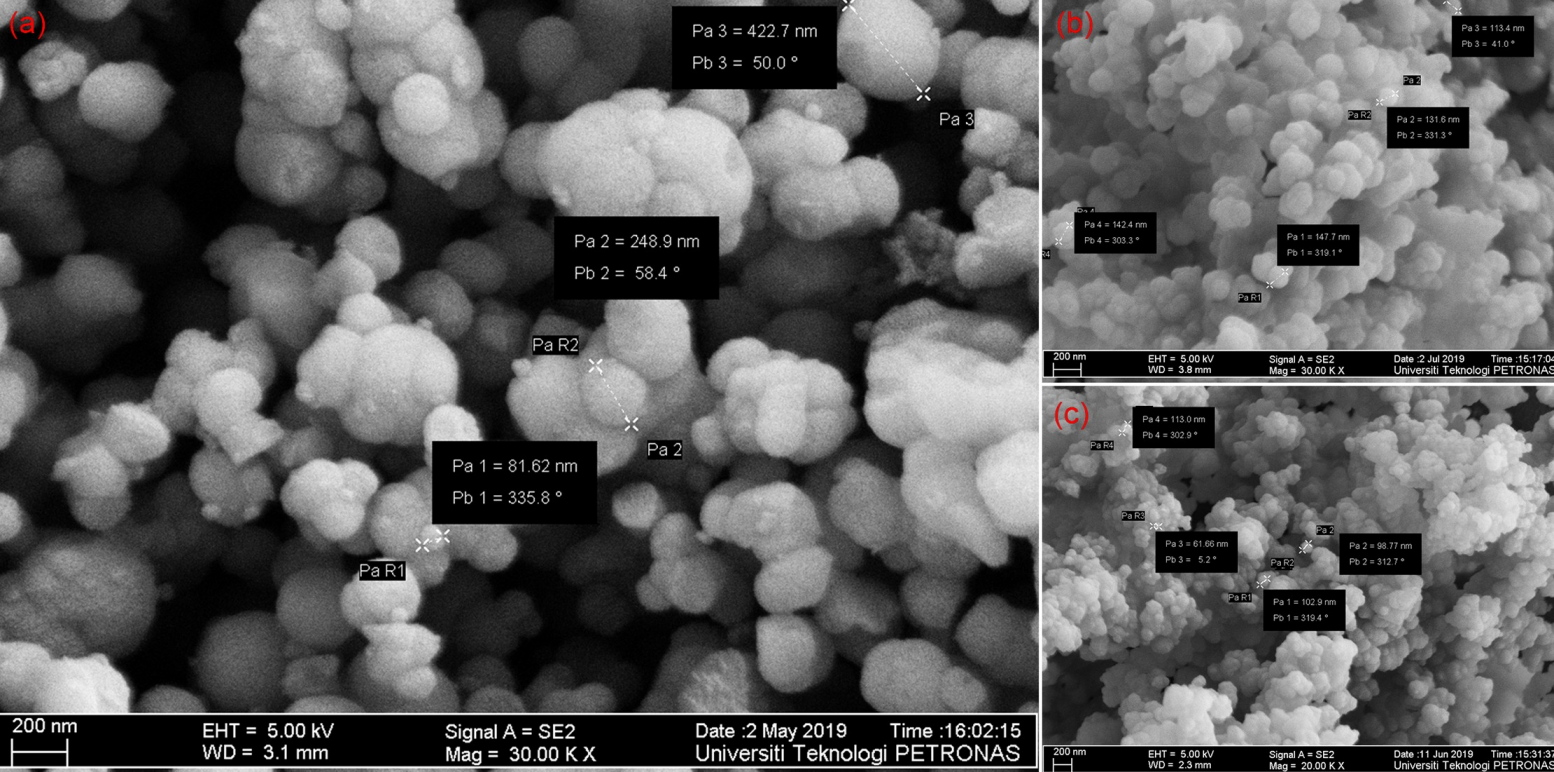
FESEM images of PEGylated silica nanoparticles synthesized via sol-gel using PEG concentration of (a) 0.1g, (b) 0.01g, and (c) 0.001g.

The typical FESEM images of PEGylated silica nanoparticles at different PEG concentration is shown in Fig 7, where the nanoparticles are spherical with the noticeable polydispersion. This reveals that PEGylated silica NPs of varying sizes can be generated by adjusting the PEG concentration. By changing the concentration of PEG in the reaction solution, nanoparticles of approximately 80–250 nm can be formed. The trend observed shows that the increase in the amount of PEG leads to a formation of bigger-sized particles. This may be the result of increased thickness in PEG shell around the silica core, leading to an increase in overall particle size. In our experiments, the amount of ammonia used in the synthesis was kept constant, as it was not observed to have a significant impact.

**Fig 8:**
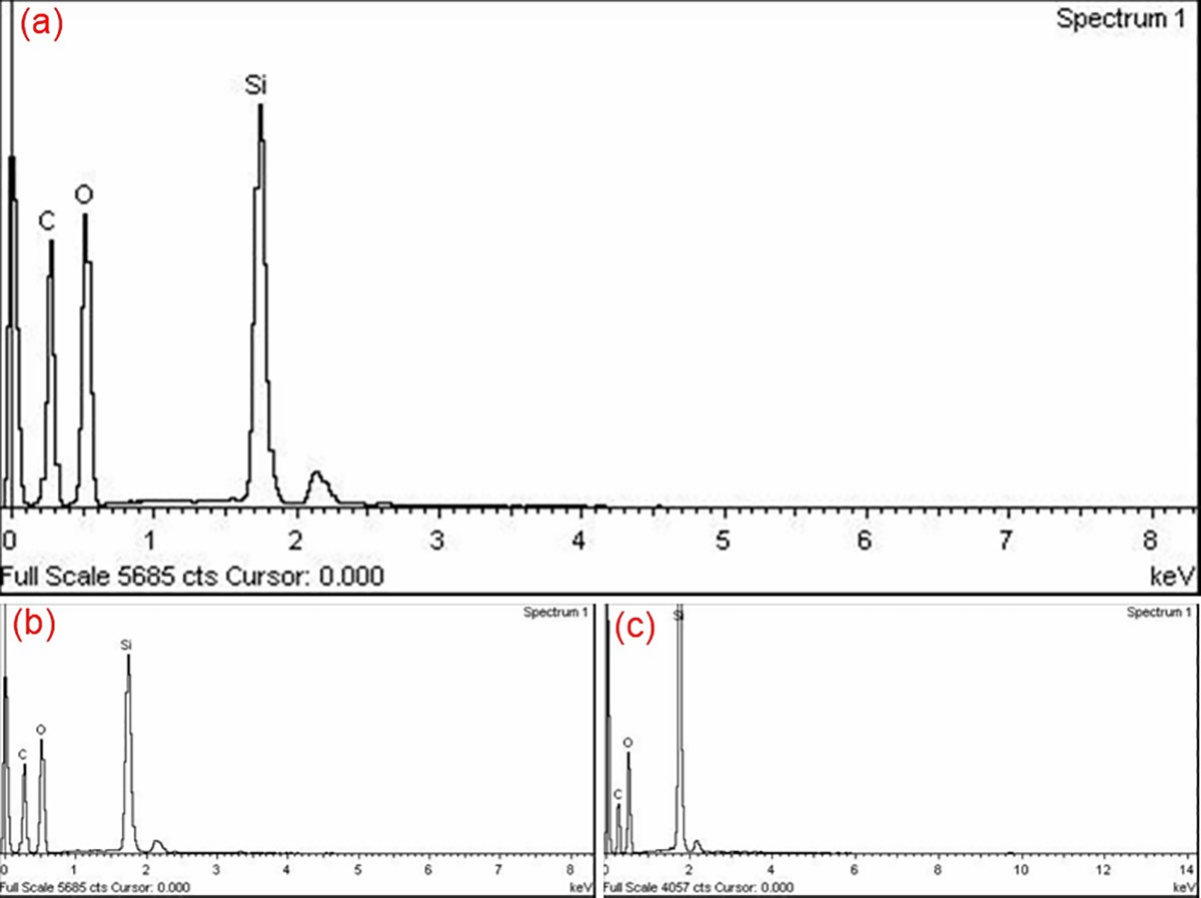
EDX spectrum of silica/PEG nanoparticles synthesized by varying PEG: a) 0.1g, b) 0.01g, and c) 0.001g.

The EDX spectra for nano samples at different concentrations of PEG are illustrated in Fig 8. The spectra of PEGylated silica nanoparticles confirm the presence of C, O, and Si in the nanoparticles. However, there is less amount of Si element for NPs sample with 0.1 gm PEG than other samples, thus yields indirect evidence for the existence of excessive amount of PEG on the surface of silica nanoparticles, leading to an increase in particle size. A similar observation was made by Timin et al. by incorporating PVP in the silica matrix [56].

**Fig 9:**
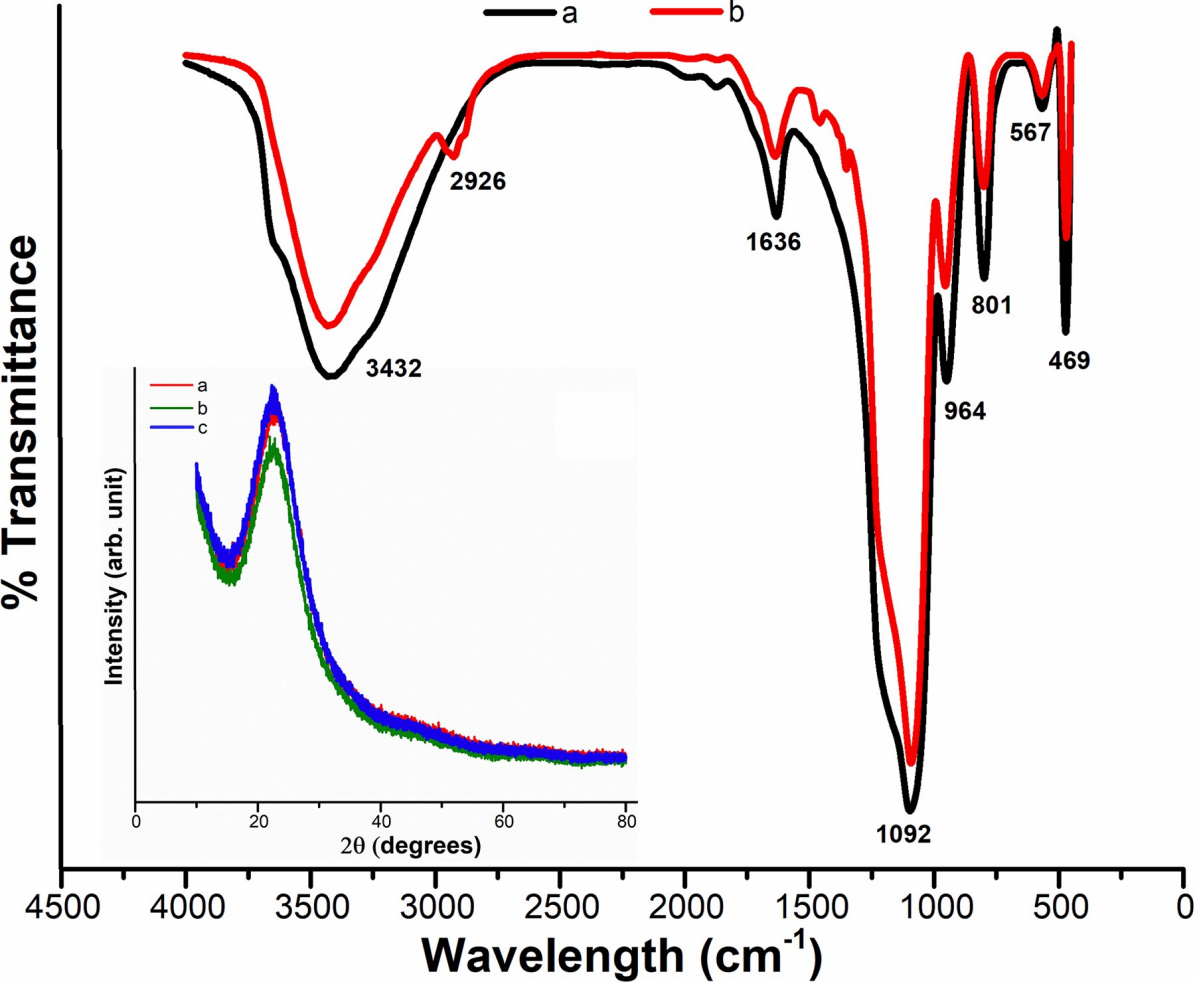
FTIR spectra of (a) as-synthesized bare nano-silica particles, in comparison with (b) PEG/silica NPs, along with XRD pattern (the insert figure) of PEGylated silica NPs at varying concentrations of a) 0.1g, b) 0.01g and c) 0.001g of PEG.

The chemical attachment of PEG to the silica NPs was indicated by FTIR, as shown in Fig 9. The very broad peak at 3432 cm^-1^ shows the presence of exchangeable protons, mainly from OH groups on the surface of the nanoparticles. The spectrum of PEG-coated silica nanoparticles provides a new absorption peak at 2926 cm^-1^, corresponding to the characteristic peaks of PEG 20000 monomethyl ether. Therefore, these peaks of PEG-coated silica nanoparticles should be from the PEG, because they were not found in the pure SiO_2_ sample, so the only distinction being the addition of PEG. Such peaks indicate the chemical binding of PEG to the silica NPs, though the transition is obscured by the contrasting peaks of the pure SiO_2_ NPs. It should also be noted here that the PEGylated silica nanoparticles were rinsed with large quantities of ethanol and water before testing, meaning that the PEG polymers, which are only physically attached on the nanoparticle surface, would actually have been washed off and the signal was only from chemically bound PEG. As demonstrated earlier in Eqs (6) through (8), the PEG polymer remains bound to the surface of the nanoparticles due to the basic condensation reaction between the silanol groups on the nanoparticle surface and the end alcohol groups on the PEG chain, which creates an ester bond (Si—O—C) [57]. Meanwhile, as seen in the wide-angle XRD pattern in Fig 9 (the insert image), a single broad peak was observed at 2θ ≈ 23° which suggests that all the samples of PEG-silica nanoparticles are amorphous and non-crystal structures were formed during the reaction phase. These findings are in accoradance with the previous studies [58,59].

The high porosity of the PEG/silica nanoparticles was verified by the measurement of the Brunauer-Emmett-Teller (BET) and nitrogen adsorption-desorption isotherms. The results reveal that the final products have a uniform pore-size of mesopores. The BET specific surface area increases with the decrease in PEG concentration as tabulated in Table 4. The surface area decreases readily due to the agglomeration, where nanoparticles join together to create a bulky structure that is larger than individual nanoparticles [54]. While the pore sizes calculated by BJH method show a respective decrement, which is consistent with FESEM observations (Fig 7). The pore size supports the IUPAC definition of pore size i.e. 2–50 nm, for mesoporosity behavior in the silica [40]. The high volume and pore size of nano-samples reveal the presence of open structures associated with agglomeration [39]. These open structures result in nanoparticles being loosely packed. Also, the observed decrease in the particle size corresponding to the decrease in PEG concentration suggests that these nanoparticles could be chemically reactive.

**Table 4:** Surface and pore analysis of silica/PEG nanoparticles.

Properties	PEG concentration (gm)		
	0.1	0.01	0.001
BET surface area (m^2^ g^-1^)	335.41	414.71	926.18
Micropore area (m^2^ g^-1^)	122.07	236.99	256.53
Micropore volume (cm^3^ g^-1^)	0.0620	0.1287	0.1313
Average pore diameter (nm)	5.69	4.39	4.23
Average particle size (nm)	8.94	7.23	3.23

### Colloidal stability of silica nanofluids

In this section, the surface-modification effect on the stability of the nano-suspension was inspected at varying pH and nanoparticle concentration.

### Influence of pH

**Fig 10:**
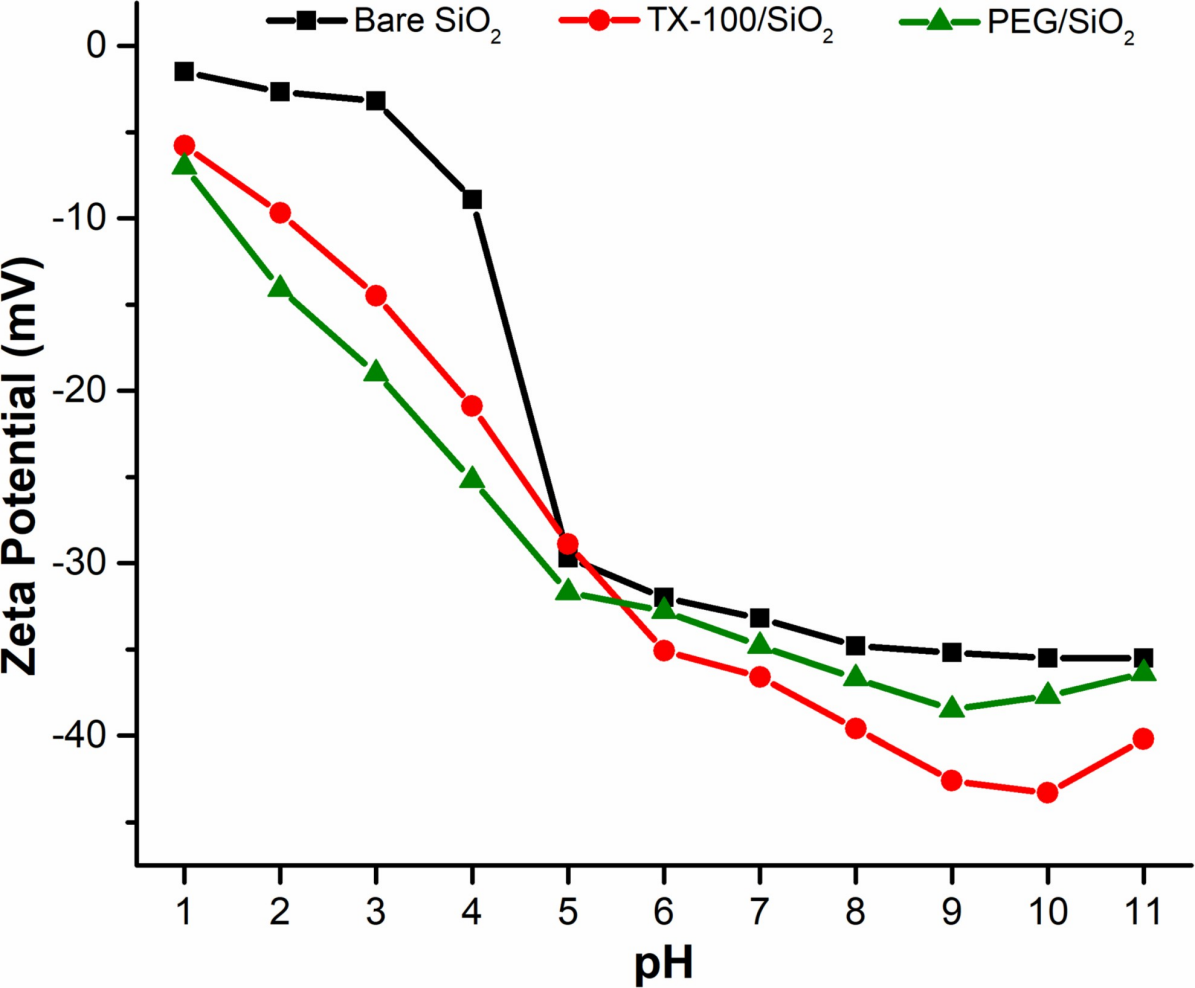
Effect of pH value on the zeta potential of silica NFs during autotitration.

The pH level is a significant factor influencing nanofluid stability. To determine the optimum pH value, autotitration was first conducted to determine the range of pH that provides relatively high zeta potential. For autotitration, 0.1 wt.% of each type of nanoparticles (bare silica, silica/TX-100, silica/PEG) were dispersed in deionized water, due to the limitation of Zetasizer at a high salt concentration of 3 wt.%. The nano-suspensions were autotitrated with acid and base over the pH spectrum of 2 to 12 as shown in Fig 10. It shows that the zeta potential of all the nano-samples in the acidic region is between -1 mV to -10 mV, which yields poor stability. However, with an increase in NaOH, the zeta potential of the SiO_2_ NFs changes and reaches to the maximum value of -43 mV at pH 9.5 for silica/PEG nanofluid. With a larger zeta potential (>30 mV), high electrical repulsion can be generated between NPs, thus limiting nanoparticle aggregation. Whereas, electrical repulsion between nanoparticles is generated at a lower zeta potential (<30 mV), which cannot prevent nanoparticles from aggregating sufficiently, leading to the low stability of nanofluids [60]. A greater electrostatic repulsion also makes the nanoparticles relatively independent, suggesting that the stability of SiO_2_ dispersion is improved at the basic pH. Hence, the three pH values of 9, 9.5, and 10 were chosen to determine the optimum pH by employing the traditional batch sedimentation approach and photographic method for analyzing settling characteristics of 0.1 wt.% silica suspensions in 3 wt.% NaCl.

**Fig 11:**
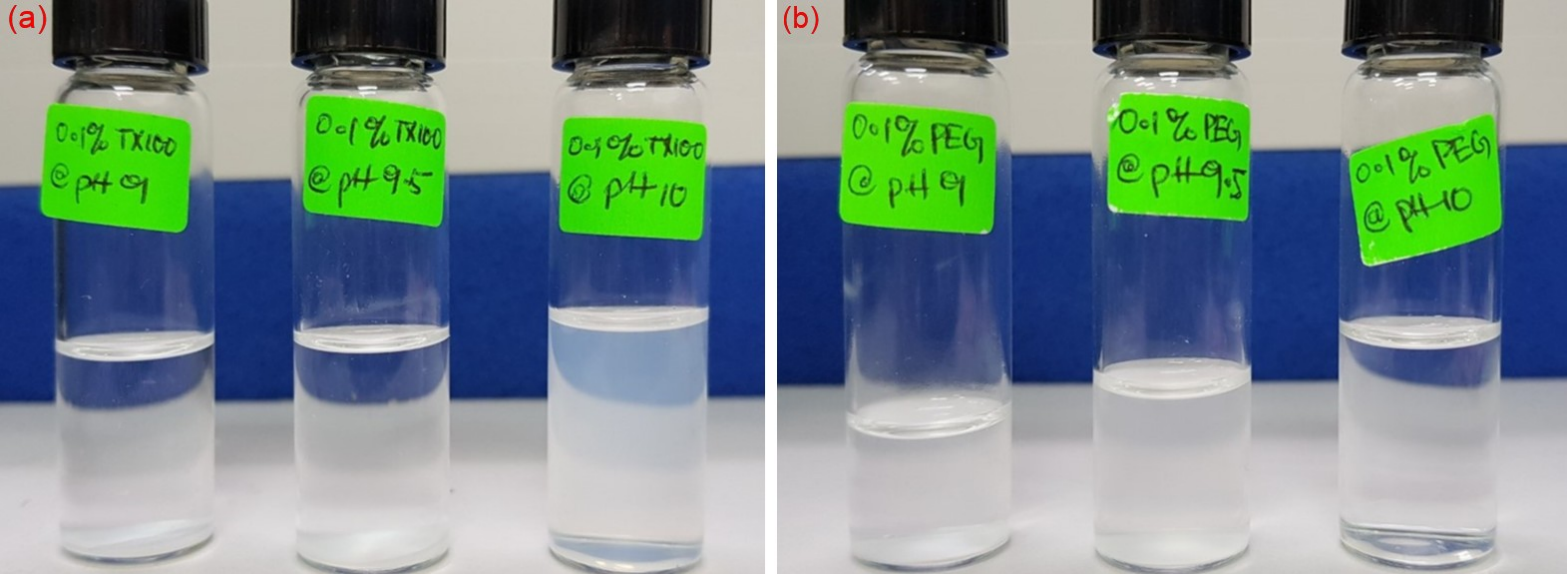
Visual monitoring of the stability of (a) TX-100/silica NFs and (b) PEG/silica NFs under different basic conditions at an optimal concentration of 0.1 wt.%.

Based on sedimentation test, the bare-silica NPs show total sedimentation for all three values of pH due to charge screening at 3 wt.% NaCl. On the other hand, in-situ modified nanoparticles show greater stability through visual monitoring (Fig 11). Silica/TX-100 NFs at pH 10 shows slight sedimentation by the end of 24 hours, which is in accordance with its relatively high zeta potential value of -38 mV. However, the suspension of silica/PEG NPs recorded a much higher value of zeta potential (-43 mV at pH 9.5), leading to no sedimentation for one whole day. This difference in zeta potential is due to the nonionic nature of TX-100 surfactant, which cannot be ionized in water and has a decreased effect on the surface charge of SiO_2_ NPs. Therefore, pH 10 and 9.5 is chosen as an appropriate condition for the preparation of the stable silica/TX-100 and silica/PEG nanofluids, respectively.

### Influence of NPs concentration

To determine the impact of NPs concentration on the stability of the in-situ modified silica nanofluid in 3 wt.% NaCl, hydrodynamic size of a series of silica NFs with various concentrations (0.05 wt.%, 0.075 wt.%, 0.1 wt.%, and 0.2 wt.%) were measured. The zetasizer is unable to determine the effective zeta potential in high salt concentration of 3 wt.% NaCl, due to the noisy phase plots. The pH levels of all nanofluids were adjusted to their corresponding optimal pH. According to Table 5, the hydrodynamic size of both silica NFs decreases with an increase in NPs concentration, until it reaches particle saturation at 0.1 wt.%. This can be the reason of charge screening at lower NPs concentration due to the presence of salt, which decreases the surface charge and agglomerates the nanoparticles. In the case of silica/TX-100 NPs, the increased concentration of NPs counterbalances the charge screening from salt to form the protecting layer by entangling the hydrophobic hydrocarbon chain of TX-100 on the surface of silica nanoparticles. Thus, increases the electrostatic repulsion between nanoparticles, which in turn enhances the dispersion stability. However, once the NPs concentration reached saturation, excessive NPs (at 0.2 wt.%) cause steric hindrance by loosely arranging the TX-100 molecules, resulting in an increase in the radius of silica nanoparticles as evident in Table 5.

**Table 5:** Hydrodynamic particle size of TX-100/silica and PEG/silica nanofluids in brine (3 wt.% NaCl) at varying weight percentage and their corresponding optimal pH.

NPs concentration (wt.%)	Hydrodynamic size (nm)		
	Bare SiO_2_	TX-100/SiO_2_	PEG/SiO_2_
0.05	7317	722.9	2653
0.075	4412	577.8	1083
0.1	3854	461.9	1025
0.2	9432	1358	1851

A similar trend is observed for silica/PEG nanofluids, where the hydrodynamic size decrease with the increase in NPs concentration, before the nanoparticles being saturated at 0.1 wt.%. This is due to the PEG adsorption that involved the modification of one end of the PEG chain to generate a PEG silane derivative for binding chemically on the surface of silica NPs. At low concentrations of nanoparticles, the difference in the thickness of the adsorption layer allows the molecules to be dispersed on the surface of the sample and attached to a small number of segments. The increase in polymer concentration induces a readjustment of the adsorbed layer structure on the molecules by straightening the previously formed bonds and exposed so that the thickness of the layer increases. At 0.2 wt.% NFs, the number of polymer molecules increases, which in turn inhibit the particle-particle interaction and form agglomerates.

### IFT reduction

The interfacial tension between crude oil and different types of silica NFs, prepared at varying nanoparticle concentrations, was measured using the spinning drop method at 7,000 rpm and room temperature. There were no visible aggregates of NPs due to the high rotational speed, and the IFT could be determined for all the samples. Table 6 shows the IFT measurement between crude oil and brine/silica NFs at varying concentrations. The IFT of the brine-crude oil system decreased from 14.3 mN/m (reference value) to a minimum value of 7.06 mN/m as the TX-100/silica NPs were introduced into the base fluid at a concentration of 0.1 wt.%. On the other hand, the IFT values at an optimal concentration of 0.1 wt.% for bare silica and PEG/silica NFs were measured to be 10.12 and 8.15 mN/m, respectively. The interfacial tension is also observed to be sensitive to nanofluid concentration, as IFT decreases with the increase in nanoparticle concentration up to 0.1 wt.%, while beyond this value, the effect of an increased concentration of nanoparticles on IFT decreases. Binks [61] also observed the shifts in the IFT as a function of NPs concentration. This change in the IFT values in correspondence to the varying concentrations of the nanofluids may be related to the nanoparticles’ surface energy, which is directly correlated with the average particle size as indicated in the following equation:

$$E=\pi r^{2}\gamma_{wo}(1\pm \cos\theta )$$ (9)

where *r* is the particle radius, *γ*_*wo*_ is the IFT between the oil/nanofluids and *θ* is the contact angle with the solid surface.

**Table 6:** Interfacial tension values measured at varying concentrations of silica nanofluids.

NPs concentration (wt.%)	Interfacial tension (mN/m)		
	Bare SiO_2_	TX-100/SiO_2_	PEG/SiO_2_
0.05	12.78 ± 1.1	8.92 ± 0.6	9.75 ± 0.6
0.075	12.44 ± 0.8	8.09 ± 0.2	9.22 ± 0.5
0.1	10.12 ± 0.5	7.06 ± 0.1	8.15 ± 0.5
0.2	10.85 ± 0.4	7.89 ± 0.5	8.80 ± 0.9

As shown in Table 5, the hydrodynamic size of surface-modified silica NPs decreases with the increase in concentration until 0.1 wt.%, and then increase again for 0.2 wt.%. The smallest hydrodynamic size of 461.9 nm was observed for 0.1 wt.% TX-100/silica, leading to an increase in surface free energy due to the high surface-to-volume ratio, and hence results in the lowest value of measured IFT among other nanofluid samples.

### Wettability alteration

**Fig 12:**
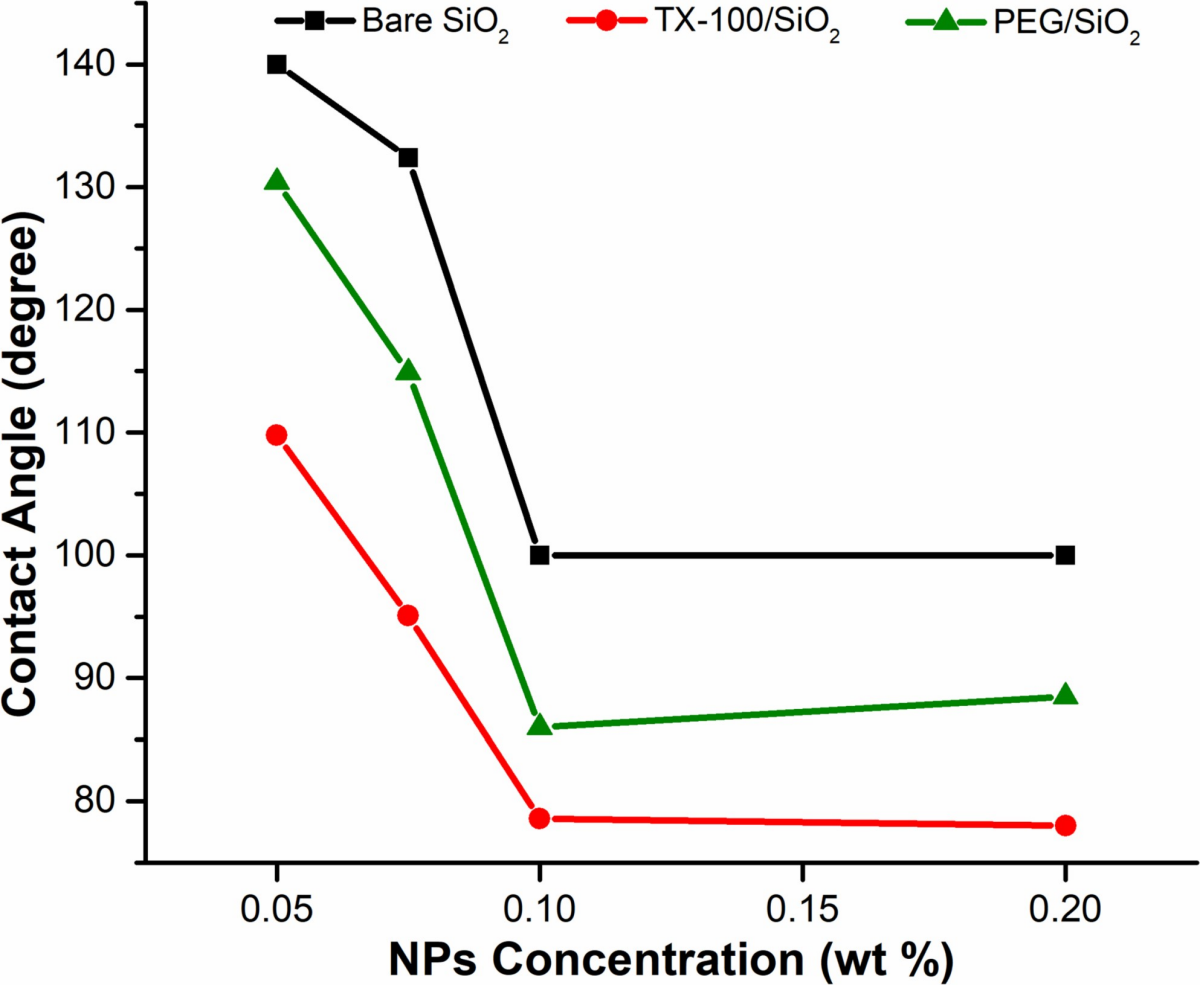
Effect of NPs concentration on the 3-phase contact angle of crude oil with the treated glass surface after being aged for 24 hours in silica nanofluids.

The impact of silica NFs on the wettability of treated (oil-wet) glass surfaces was studied after 24 hours of soaking at room temperature in the suspension of varying concentrations of surface-treated silica NPs. As shown in Fig 12, the contact angle was slightly decreasing up to 0.075 wt.% NPs concentration. However, at 0.1 wt.%, a noticeable reduction from 130.4° to 78° occurred for silica/TX-100 nanofluid, suggesting a rapid change in the wettability of glass surface towards water wetness. While, the wettability changes to intermediate for 0.1 wt.% bare-silica and silica/PEG nanofluids with a contact angle value of 100° and 86°, respectively. The surface water wetness increases due to the strong electrostatic repulsion between nanoparticles, which allows nanofluid to spread along the rock surface, resulting in a decreased contact angle. However, the wettability remains almost unchanged despite a further increment in NPs concentration to 0.2 wt.%, which shows that, besides undesirable instability of the suspension, highly concentrated nanofluid has no major impact on changing the wettability towards the favorable state. This trend of change in wettability is in accordance with the previous studies, where the surface-coated silica nanofluids have altered the rock wettability to the strong water-wet state, demonstrated by a decrease in the contact angle [9,62].

Table 7 presents the final values of contact angles corresponding to the oil-wet state, which incredibly restored to the initial (untreated) water-wet state of the glass surface due to the synergistic influence of silica NPs and salinity. The structural disjoining pressure is the possible mechanism behind the observed trend, which was simultaneously imposed by the influence of NPs as well as seawater salinity, contributes to a reduction of contact angle. In this mechanism, negatively charged nanoparticles scatter around oil droplet lodged on the glass surface, where the water-oil interface under the oil droplet has a negative charge, except at the high pH and ionic strength caused by the formation brines [63] and/or in highly acidic crude oil [64]. This results in the repulsion forces between the interfaces that lead to the separation of oil droplets from the solid surface by nanoparticles, and consequently, shifts the wettability of the surface from oil-wet to water-wet. Additionally, the higher uniformity of silica/TX-100 NPs compared to that of other synthesized NPs provides better and effective dispersion in brine (see in Table 5), which leads to better interaction between surfactant-treated silica NPs and the anionic acidic components of the crude oil adsorbed on the glass surface, and thereby increases the wettability alteration (ion-binding mechanism).

**Table 7:** Contact angle values of crude oil on treated (oil-wet) glass surface after being aged in the nanofluid for 24 hours.

Nanofluids	Contact angle (degree)	
	Before aging	After aging
Bare SiO_2_	140 ± 5.5	100 ± 3.8
TX-100/SiO_2_	130 ± 3.2	78 ± 2.0
PEG/SiO_2_	132 ± 4.0	86 ± 3.1

**Fig 13:**
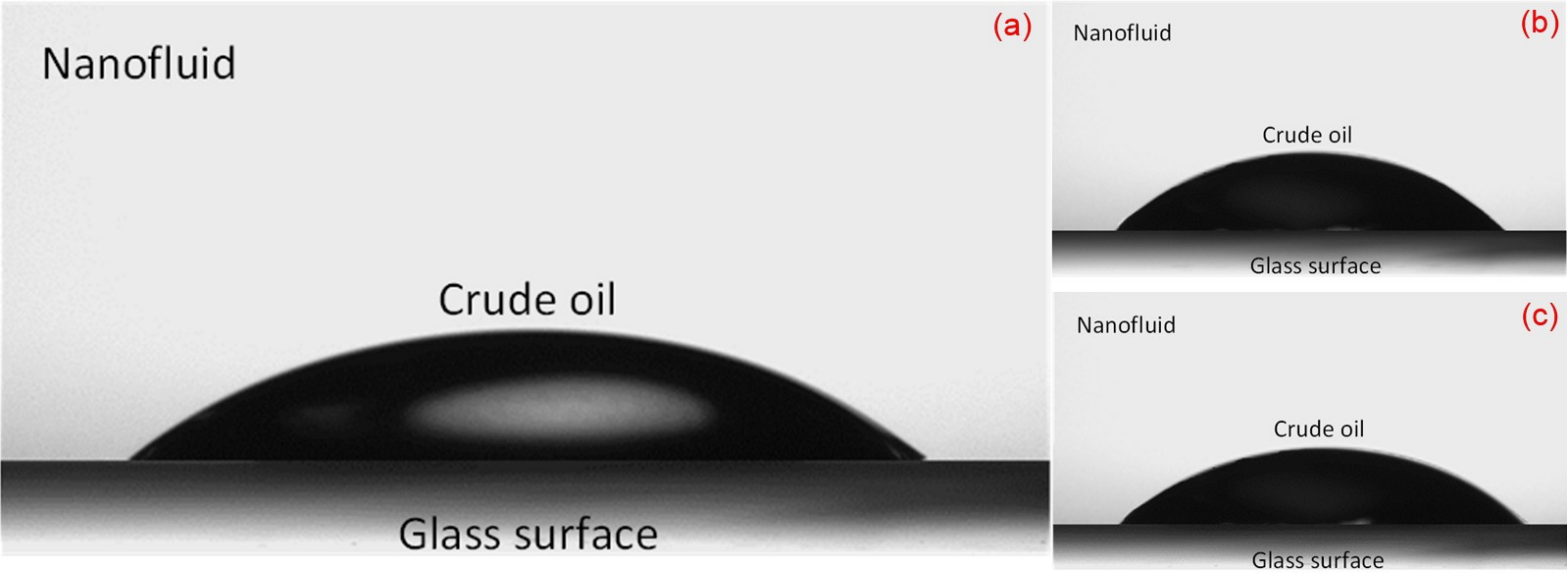
Close-up views of three-phase contact angle before aging (oil-wet glass surface) in the (a) bare SiO_2_, (b) TX-100/SiO_2_, and (c) PEG/SiO_2_ nanofluids.

**Fig 14:**
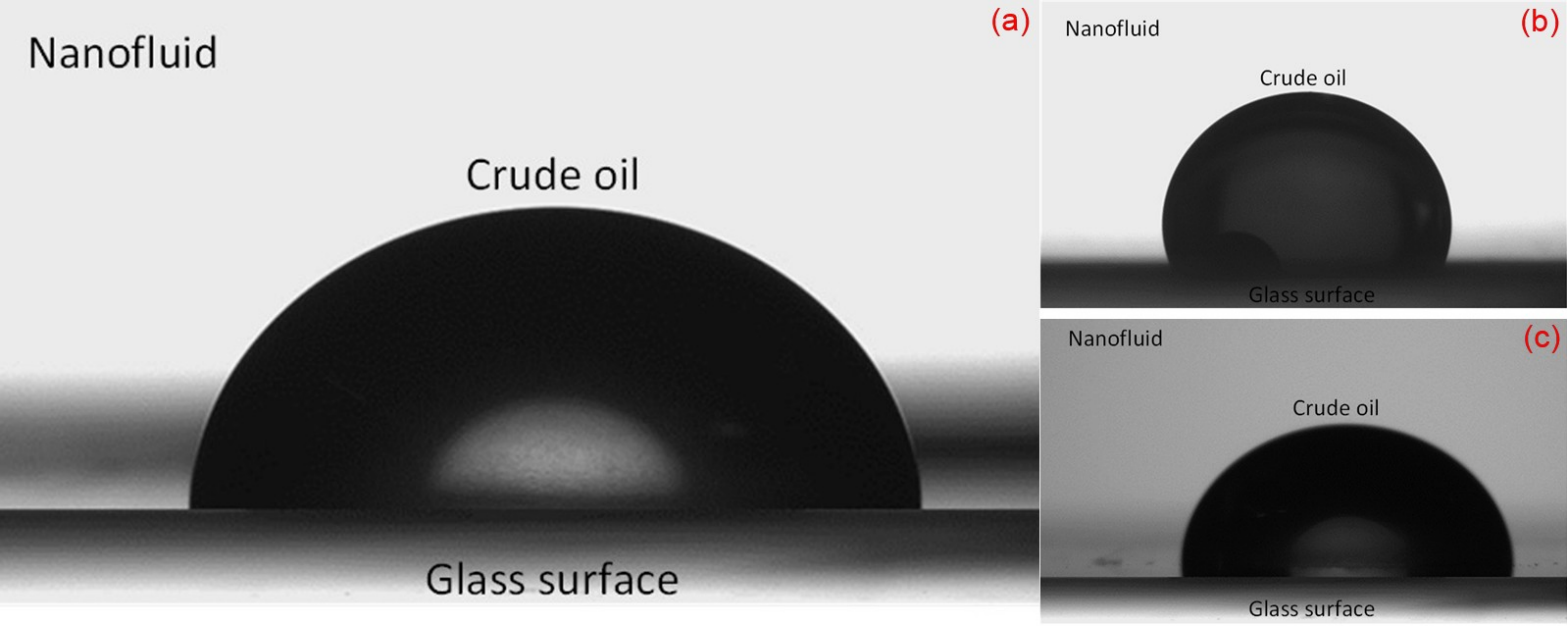
Close-up images of contact angle after being aged in the (a) bare SiO_2_, (b) TX-100/SiO_2_, and (c) PEG/SiO_2_ nanofluids.

The images of the oil droplet profile on the treated glass surface, before and after being aged with the different types of silica nanofluid, are shown in Figs 13 and 14. The surface energy, as mentioned earlier, may also have induced a shift in the contact angle corresponding to particle size. The surface-free energy increased correspondingly to the surface-to-volume ratio for smaller nanoparticles (e.g. silica/TX-100 NPs), which leads to a decrease in the contact angle. On the other hand, the opposite occurred for the bigger NPs. The similar trend observed by Vafaei et al. also revealed that the smaller nanoparticles were more effective in reducing the sessile droplet contact angle [65].

## Conclusion

The effect of surface-modification on the size and the morphology of silica NPs, as well as on oil recovery mechanisms (IFT and wettability) have been studied in this work. Three types of silica NPs, including bare-SiO_2_, TX-100/SiO_2_, and PEGylated SiO_2_, were produced by sol-gel method with reverse micelle microemulsion and were characterized by FESEM, EDX, XRD, FTIR, and BET. The FESEM images showed the formation of well-defined spherical nanoparticles with the minimum particle diameter between 13 to 27 nm for TX-100, while EDX and FTIR pattern verifies the successful surface-modification of SiO_2_ nanoparticles. In XRD spectra, the amorphous structure of the prepared nanoparticles has been confirmed by a single wide peak. On the other hand, BET specific surface area decreases with the increase in Triton X-100 as well as PEG concentration due to the agglomeration. The effects of pH and NPs concentration on the hydrodynamic size were also thoroughly investigated. The optimal stability conditions were obtained at 0.1 wt.% SiO_2_ NPs at a basic pH of 10 and 9.5 for TX-100/SiO_2_ and PEG/SiO_2_ nanofluids, respectively. To study the effect of surface-modification and particle size on oil recovery mechanism, IFT and contact angle measurements were performed. The minimum IFT value, 7.06 mN m^-1^, and the biggest change in the three-phase contact angle, 48%, were achieved by the TX-100 coated SiO_2_ NPs. The contact angle of the treated glass surface after soaking in different concentrations of silica nanofluid reveals a significant change of wettability with the increase in NPs concentration. This was attributed to the change in wettability state of the glass surface from oil-wet to water-wet, caused by the uniform and effective dispersion of spherical TX-100/SiO_2_ NPs that provides better interaction with crude oil components as well as the solid surface.

## Supporting information

S1 File EDX spectrum images. Images used to perform compsitional analysia of bare and surface modified silica NPs.
(RAR)

S2 File XRD analysis. The raw data files employed to deteremine XRD pattern and crystalline structure.
(RAR)

S3 File BET analysis. The data used to determing porosity and surface area of all types of as-synthesized SiO_2_ NPs.
(RAR)

S4 File Hydrodynamic size. The raw data files used to calculate hydrodynamic size of surface-modified SiO_2_ NPs.
(RAR)
